# Improving congenital heart disease imaging using 3d whole-heart dual-phase MRI

**DOI:** 10.1186/1532-429X-13-S1-P1

**Published:** 2011-02-02

**Authors:** Tarique Hussain, Dirk Lossnitzer, Sergio Uribe, Hannah Bellsham-Revell, Isra Valverde, Reza Razavi, Phillip Beerbaum, Aaron Bell, Rene Botnar, Tobias Schaeffter, Gerald Greil

**Affiliations:** 1 King's College London, London, UK; 2 Pontificia Universidad Catolica de Chile, Santiago, Chile

## Introduction

A single-phase 3d steady-state-free precession whole heart approach with respiratory navigator gating and ECG triggering (3d SSFP) is now frequently used for diagnostic imaging in children with congenital heart disease (CHD). However, certain cardiac structures may be better assessed during the systolic and others in the diastolic rest period. Therefore, a previously described dual phase 3d-SSFP whole heart sequence may offer clinical benefit in these patients. (*Uribe et al 2008*)

## Purpose

To determine whether 3d-dual phase imaging for CHD offers benefits in demonstrating or measuring cardiovascular structures.

## Methods

50 consecutive children with CHD underwent 3D SSFP dual-phase imaging. All cardiac chambers and great vessels were analyzed for contrast-to-noise ratio (CNR) and image quality (IQ) (consensus reading by two independent observers; grade 0=non diagnostic; 1 to 4=diagnostic, *McConnell et al 1997*). Twelve patients were referred for RVOT assessment. Dimensions were measured at valvar, supravalvar and pre-bifurcation levels. CNR, image quality and RVOT measurements were compared between systole and diastole.

## Results

**Figure 1:**
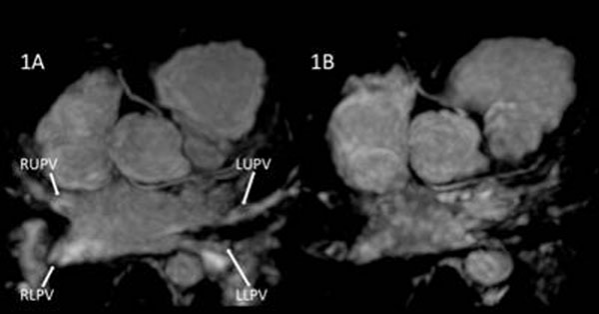
**A 6-yr-old patient, status post repair of Tetralogy of Fallot.** Identical formatting showing thick slab (1.6cm) depicting pulmonary veins. Systolic pulmonary vein imaging (A) has superior IQ (grade 2 vs. 1) and CNR (5.3 vs. 4.9) to the diastolic image (B). (RUPV – right upper pulmonary vein; RLPV – right lower pulmonary vein; LUPV – left upper pulmonary vein and LLPV – left lower pulmonary vein)

**Figure 2:**
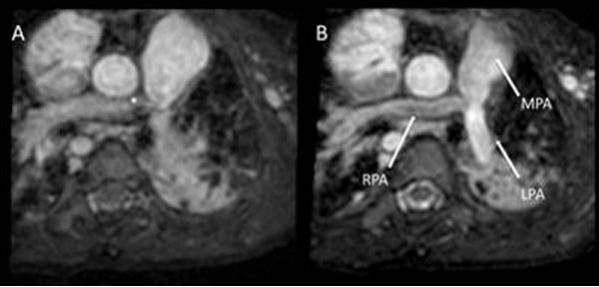
**A 4-year-old patient with complex bifurcation stenosis of pulmonary artery and branches (arrows).** This is only appreciated in the diastolic image (right) and not in the systolic image (left), which shows image disturbance due to flow turbulence (asterix). (RPA – right pulmonary artery; LPA – left pulmonary artery and MPA – main pulmonary artery)

50 children (26 male, mean age 4yrs 10months, range 5days to 18yrs) underwent 3d dual-phase SFFP imaging. The mean HR was 90 bpm (56 to 139). Dual phase yielded diagnostic imaging for all chambers and great vessels in 48 cases. Systolic imaging alone was diagnostic in 45 cases and diastolic imaging in 47 cases. CNR (paired t-test) and IQ (Wilcoxon Signed Ranks test) were significantly (p<0.05) higher in systole for the pulmonary veins (figure 1), left atrium and both ventricles. Conversely, diastolic imaging had significantly higher CNR values for the aorta and superior cavo-pulmonary (Glenn) anastomosis. IQ was significantly higher in diastole for the branch pulmonary arteries and for arterial stenoses (e.g. coarctation, pulmonary artery stenosis; figure 2). Cross sectional measurements of the RVOT were significantly larger in systole at all 3 levels (valve 2.9 vs. 2.7cm^2^; supravalvar 3.2 vs. 2.7cm^2^ and bifurcation 3.3 vs. 2.5cm^2^: p<0.05 by paired t-test, max. difference to diastole 50%).

## Conclusion

Dual phase 3D SSFP imaging improved the success rate of diagnostic quality imaging compared to single-phase imaging. Cardiac chambers and pulmonary veins are better imaged in systole possibly due to improved blood exchange. Less blood flow turbulence may have improved branch pulmonary arteries or arterial stenosis imaging in diastole. Our data confirms that under-sizing of RVOT stent-valve would have occurred if diastolic imaging were used to guide intervention.

